# Gross alpha and gross beta activities in selected marine species in Vietnam

**DOI:** 10.1007/s11356-020-09874-y

**Published:** 2020-07-01

**Authors:** Hao Duong Van, Huy Le Luong, Chau Nguyen Dinh, Duong Nguyen Thanh, Miklós Hegedűs, Anita Csordás, Tibor Kovács

**Affiliations:** 1 Institute of Research and Development, Duy Tan University, Da Nang, 550000 Vietnam; 2 AGH University of Science and Technology (AGH UST), Krakow, Poland; 3 Hanoi University of Mining and Geology (HUMG), Hanoi, 100000 Vietnam; 4 Institute of Radiochemistry and Radioecology, University of Pannonia, Veszprém, Hungary

**Keywords:** Gross alpha and beta activities, Ingestion dose, Seafood, Radionuclides, Marine pollution

## Abstract

The measured gross alpha and gross beta activities in the edible muscle tissues of eleven selected marine species along the coast of North Vietnam varied from 10.2 ± 1.5 to 73.2 ± 8.1 Bq/kg (wwt) and from 10.6 ± 0.4 to 68.8 ± 2.8 Bq/kg (wwt), respectively. The lowest gross alpha activity was recorded for bigfin reef squid (*Sepioteuthis lessoniana*) as a result of its carnivorous diet, and the highest alpha activity was noted for blood cockle (*Anadara granosa*) as a result of its omnivorous diet. However, the gross beta activities in both carnivorous and omnivorous species were similar. The highest and lowest gross beta activities were observed for narrow-barred Spanish mackerel (*Scomberomorus commerson*) and for bigfin reef squid and squid (*Teuthida*), respectively. All three aforementioned species have carnivorous diets. The calculated annual committed effective dose resulting from the consumption of 25 kg of muscle tissue per year varied from 192 to 1375 μS with an average of 689 μS.

## Introduction

Radionuclides of various origins are present in seawater, and some elements behave conservatively and remain in their soluble form in water, whereas others are insoluble, or adhere to particles, and sooner or later are transferred to marine sediments (IAEA 2005). In the marine environment, ^238^U, ^210^Pb, ^210^Po, and ^40^K are the most abundant natural radionuclides and are ultimately derived from the weathering of rocks or fallout from the atmosphere in the form of rain and are transported by river discharge (Cochran 1982; Chen et al. 2016; Filizok and Uğur Görgün 2019; Mohan et al. 2019; Peng et al. 2019). Three main sources of isotopes of artificial radionuclides exist, namely, nuclear weapons testing, the nuclear accidents at Chernobyl as well as Fukushima, and waterborne discharges from nuclear reprocessing plants (Beresford et al. 2020; Hirose et al. 1999; Kaizer et al. 2017; Kato et al. 2018; Kawamura et al. 2017; McKenzie and Dulai 2017; Nishikiori and Suzuki 2017; Ogata 2013; Ramzaev et al. 2008; Savino et al. 2017; Vlasova et al. 2015). Other sources contribute less contamination, e.g., ocean dumping of nuclear waste, routine discharges from nuclear power plants, sunken nuclear submarines, lost satellites as well as nuclear weapons, and the use of radioisotopes in medicine, industry, and science (IAEA 2005).

Several research projects concerning radiation activity, namely, that of ^40^K, ^238^U, ^232^Th, ^137^Cs, ^134^Cs, ^210^Po, and ^210^Pb concentrations in marine organisms and fish, are ongoing (Ababneh et al. 2018; Alam et al. 1995; Szefer et al. 1990). Specifically, Nandhakumari et al. (2014) studied the radioactivity content in sediment, water, and fish collected from the Rajakkamangalam Estuary of Kanyakumari District in the state of Tamil Nadu, India. Mean gross alpha and beta activity concentrations of 57.38 Bq/kg and 123.67 Bq/kg were measured, respectively (NandhaKumari et al. 2014). Zorer and Öter (2015) reported the results of their evaluation concerning gross radioactivity in foodstuffs. In their study, food items were divided into eight groups, and the levels of gross alpha and gross beta radioactivity varied tremendously from 70 to 10,885 Bq/kg and from 132 to 48,285 Bq/kg on dry mass basis, respectively. The average gross alpha and gross beta activities of fresh fish were reported to be 625 Bq/kg and 2863 Bq/kg, respectively, while for salted fish, the average gross alpha activity was not detected, but the average gross beta activity was 1554 Bq/kg (Zorer and Öter 2015). It must be noted that gross alpha and beta measurements have some limitations; depending on the measurement method, they cannot be easily compared, and certain radionuclides might not be measured (Jobbágy et al. 2014). In Vietnam, radioactivity in food has rarely been investigated (Van et al. 2018), and no baselines for radioactivity levels are available. Therefore, the objectives of this study are to (i) determine gross alpha and gross beta activities in different selected marine species in the East Sea of Vietnam and (ii) estimate the annual committed effective dose following the consumption of marine species.

## Experimental study

### Sampling and sample preparation

The eleven representative samples of seafood collected represent the most commonly consumed marine species in Vietnam, namely, Lyrate hard clam (*Meretrix lyrata*), silver pomfret (*Pampus argenteus*), narrow-barred Spanish mackerel (*Scomberomorus commerson*), shortfin scad (*Decapterus* sp.), Indian mackerel (*Rastrelliger kanagurta*), blood cockle/granular ark clam (*Anadara granosa*), giant tiger prawn (*Penaeus monodon*), tuna (*Thunnini*), squid (*Teuthida*), bigfin reef squid (*Sepioteuthis lessoniana*), and groupers (*Epinephelinae*). The local names, English names, and scientific names of each sample collected in the study according to Froese and Pauly (2019), Palomares and Pauly (2019) and also their feeding habits are listed in Table 1. The trophic levels presented in Table 1 were taken from the databases FishBase and SealifeBase and Pinnegar et al. (2003).

**Table 1:** The marine organisms in this study and their feeding habits

Local name	English name	Scientific name	Feeding habits	Trophic level
Cá Song	Groupers	*Epinephelus* sp.	Carnivores: crustaceans, octopodes, young sea turtles, other fish	(3.7)
Cá Ngừ	Tuna	*Thunnus* sp.	Carnivores: fishes and invertebrates	(4.5)
Cá Nục	Shortfin scad	*Decapterus* sp.	Carnivores: planktonic crustaceans and fishes	(3.4)
Mực Ống	Squid	*Loligo* sp.	Carnivores: other crustaceans and fish	(3.8)
Mực Lá	Bigfin reef squid	*Sepioteuthis lessoniana*	Carnivores: other crustaceans and fish	(4.0)
Cá Thu	Narrow-barred Spanish mackerel	*Scomberomorus commerson*	Carnivores: small fishes	(4.5)
Cá chim trắng	Silver pomfret	*Pampus argenteus*	Carnivores: Crustacea, Bacillariophyta, Mollusca	(3.3)
Cá Bạc Má	Indian mackerel	*Rastrelliger kanagurta*	Omnivores: macro planktons, crustacean, mollusks	(3.2)
Con Ngao	Lyrate hard clam	*Meretrix lyrata*	Omnivores: organic particles, planktons	(2.2)
Sò huyết	Granular ark/blood cockle	*Tegillarca granosa*	Omnivores: planktons, organic particles	(2.0)
Tôm sú	Giant tiger prawn	*Penaeus monodon*	Omnivores: phytoplankton, zooplankton, crustaceans, detritus, mollusks, fish parts, and mud	(3.4)

In the laboratory, the studied samples were washed with distilled water three times. The samples were separated into edible muscle tissues (which were used to assess the gross alpha and gross beta activities humans are exposed to following their consumption) and all other components (bones and hard parts). The edible muscle tissues were weighed and oven-dried at 90 °C to a constant weight and then powdered, homogenized, and reweighed to determine the dry mass to wet mass ratio. Next, the powdered samples were wet-digested using a mixed solution of HNO_3_ and HCl (1:3) followed by the addition of H_2_O_2_ until the digestion was complete. Following digestion, the samples were evaporated, and the precipitates that remained were used in the next step. A certain amount of residue from each sample was used for each measurement and spread onto the surface of the measuring trays. To ensure accurate counting and measurement stability, the density of gross α and gross β activities was 0.05 mg/mm^2^ and 0.10 mg/mm^2^, respectively. The size of the trays in this study was 25 mm, so the calculated masses of residues from each sample used to measure gross alpha and gross beta activities were controlled at 98 mg and 196 mg ±5%, respectively. To subtract the background radiation, a background radiation sample was also prepared; moreover, for the purpose of calibration, standard samples were measured. In order to maintain the counting efficiency of the instrument and avoid self-absorption by the samples from influencing the measurement results which are affected by the density and mass thickness of the samples, the masses of the samples of residues were calculated according to the following formula (Gorur and Camgoz 2014):

1 $$ \mathrm{M}={\mathrm{T}}_{\mathrm{a}}\ast \mathrm{A} $$

where *M* denotes the mass of the sample of residue (mg), *T*_a_ represents the mass thickness of the sample of residue (mg/mm^2^), and *A* stands for the effective measurement area of the sample of residue (mm^2^).

### Experimental instrument

The prepared samples were counted to determine the gross alpha and gross beta activities using a Canberra LB4100 low-background gas proportional counter (Canberra Company, USA) calibrated with ^241^Am and ^90^Sr standard surface sources. Low background count rates of 0.10 cpm and < 0.93 cpm for gross alpha and gross beta activities were used, respectively, using a gas proportional counter with a gas composition of 10% methane + 90% argon. All the studied background and standard samples were measured over 86,400 s, and alpha and beta efficiencies of 20–25% and 30–40% were determined, respectively.

The minimum detectable activity of the instrument was determined in accordance with research conducted by Janković et al. (2012) and Turgay et al. (2015), which are based on the article of Currie (1968) as follows:

2 $$ \mathrm{MDA}\left( Bq/l\right)={L}_d/{V}^{\ast }{T}^{\ast }{\varepsilon}^{\ast }60 $$

where *L*_d_ = 2.71 + 4.65√(Bc*T), *V* denotes the volume of the measured sample (liter), *T* represents the measurement time (min), *ε* stands for the detection efficiency, and Bc is the background count rate (cpm). The minimum detectable activity of the measurement system was calculated to be 1.6 mBq L^−1^ and 1.4 mBq L^−1^ for gross alpha and gross beta activities, respectively.

## Results and discussion

The gross alpha and gross beta activities in selected marine organisms are presented in Table 2. The results show that the dry mass to wet mass ratios of the marine organisms ranged from 0.12 to 0.30 with an average of 0.23 ± 0.06. The mean gross alpha and gross beta activities varied over a wide range from 10.2 and 10.6 Bq/kg (wwt) to 73.2 and 68.8 Bq/kg (wwt) for alpha and beta, respectively.

**Table 2:** Dry mass to wet mass ratio and gross alpha and gross beta activities in selected marine organisms

Scientific name (*n* = 5)	Dry/wet ratio	Gross alpha (Bq/kg wwt)	Gross beta (Bq/kg wwt)
*Epinephelinae*	0.30	21.6 ± 1.7	51.1 ± 2.2
*Thunnini*	0.30	61.0 ± 6.8	65.5 ± 3.4
*Decapterus scombrinus*	0.25	24.2 ± 2.0	38.3 ± 2.0
*Teuthida*	0.14	43.0 ± 4.8	19.1 ± 0.8
*Sepioteuthis lessoniana*	0.12	10.2 ± 1.5	10.6 ± 0.4
*Scomberomorus*	0.25	13.3 ± 0.7	68.8 ± 2.8
*Pampus argenteus*	0.25	23.4 ± 1.8	39.0 ± 2.0
*Rastrelliger kanagurta*	0.27	29.7 ± 2.4	62.4 ± 3.0
*Veneridae*	0.27	58.5 ± 5.3	47.8 ± 1.8
*Anadara granosa*	0.17	73.2 ± 8.1	42.1 ± 2.4
*Penaeus monodon*	0.21	43.7 ± 5.5	36.4 ± 1.8
Minimum	0.12	10.2	10.6
Maximum	0.3	73.2	68.8
Average value	0.23	35.6	43.7

The mean gross alpha and gross beta activities in the 11 marine species investigated were 35.6 Bq/kg (wwt) and 43.7 Bq/kg (wwt), respectively. The measured gross beta activity in the marine creatures exceeded 10 Bq/kg. The gross beta activity was greater than the gross alpha activity, which was also observed in fish samples from Lake Van, Turkey (Erenturk et al. 2014), because among the naturally occurring gamma-emitting radionuclides, the concentrations of radioactive potassium (^40^K) was the highest (Carvalho et al. 2011).

The activity is much lower in bigfin reef squid (*Sepioteuthis lessoniana*) with gross alpha and gross beta activities of 10.2 Bq/kg (wwt) and 10.6 Bq/kg (wwt), respectively, which is related to its carnivorous feeding type. The diet of bigfin reef squid consists of crustaceans and fish. The gross alpha activity of species that belong to the carnivorous feeding type ranges from 10.2 ± 1.5 to 61.0 ± 6.8 Bq/kg (wwt) (groupers, tuna, shortfin scad, squid, bigfin reef squid, narrow-barred Spanish mackerel, silver pomfret) while that of species belonging to the omnivorous feeding type ranges from 29.7 ± 2.4 to 73.2 ± 8.1 Bq/kg (wwt) (India mackerel, Lyrate hard lam, blood cockle, giant tiger prawn). The variation in the gross alpha activity of various species of marine food collected from the same coastal region could be attributed to the metabolism, feeding type, and size of the species. Some studies showed that lower activities of radionuclides detected in carnivorous marine fish could be related to their slower rate of metabolism (Mat Çatal et al. 2012; Ababneh et al. 2018). The highest gross alpha activity of 73.2 ± 8.1 Bq/kg (wwt) was measured in blood cockle (*Anadara granosa*), an omnivorous bottom feeder whose diet consists of plankton and organic particles. Given the feeding type of blood cockle, radionuclides probably have a high degree of association with organic matter. Its environment, plankton, and bottom-feeding habits were suggested to lead to the accumulation of and significantly contribute to the relatively high radionuclide activity (Ababneh et al. 2018; Aközcan 2013; Chen et al. 2016; Raja and Shahul Hameed 2010; Štrok and Smodiš 2011). The relatively high gross alpha activities of the omnivorous species Lyrate hard clam and giant tiger prawn (*Penaeus monodon*) are 58.5 ± 5.3 Bq/kg (wwt) and 43.7 ± 5.5 Bq/kg (wwt), respectively. Bivalve mollusks, e.g., blood cockle and Lyrate hard clam, are capable of accumulating contaminants in biological systems, so they are used as indicators of pollution not only in terms of radionuclides but also heavy metals as well as pesticides (Forester 1980) and accumulate much more radiation activity than other marine organisms (Musthafa and Krishnamoorthy 2011). However, the gross alpha activity in tuna (*Thunnini*), a carnivorous species, was close to the highest value 73.2 ± 8.1 Bq/kg (wwt). It should be noted that gross alpha activity not only depends on feeding habits, the rate of metabolism, and the environment but also on the size of the species, its age, and other parameters, e.g., the depth and temperature of the water. The tuna in this study was the biggest species.

**Fig. 1:**
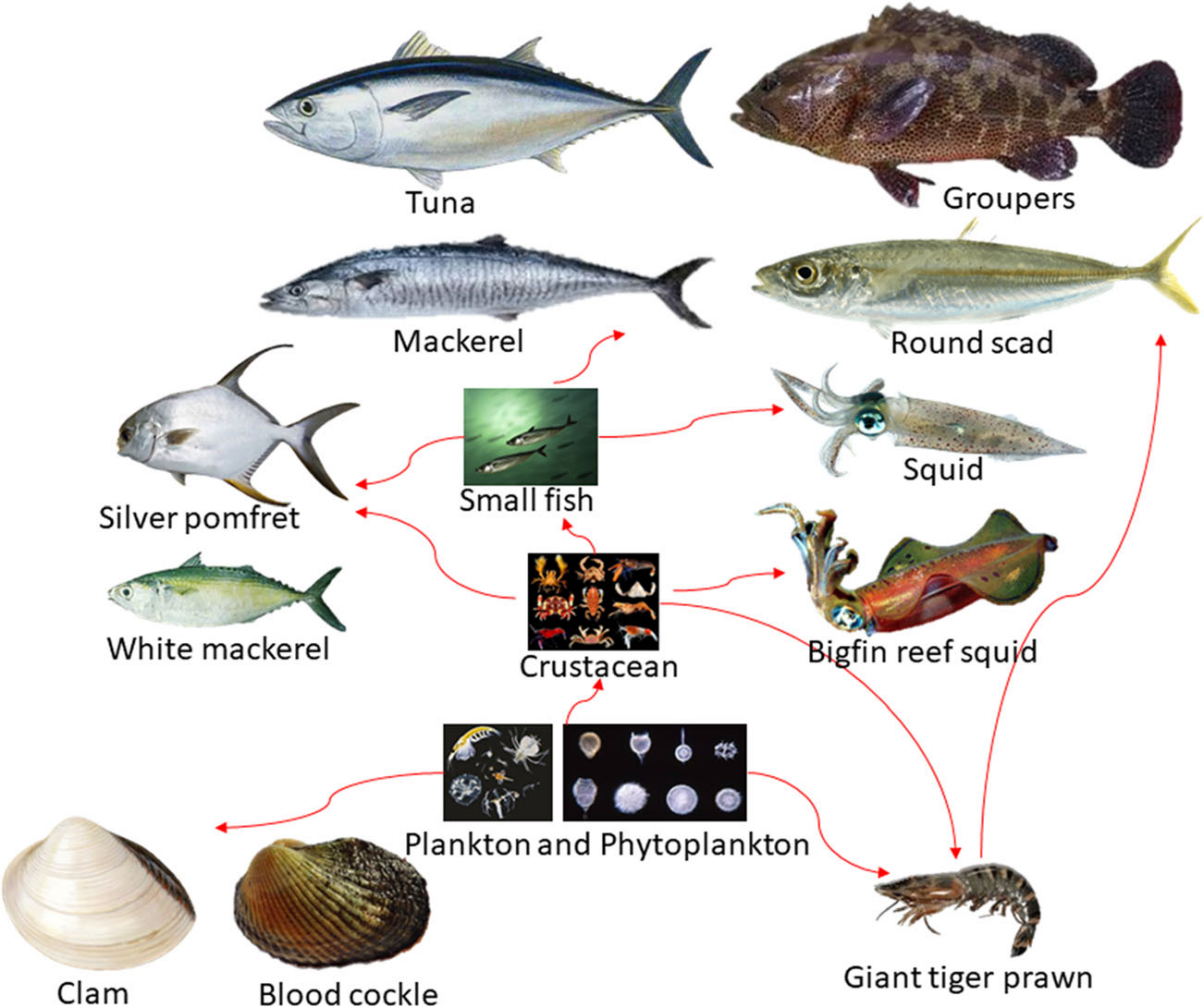
Food chain in a marine ecosystem

The gross beta activity of species that belong to the carnivorous feeding type ranges from 10.6 ± 0.4 to 68.8 ± 2.8 Bq/kg (wwt) while that of those belonging to the omnivorous feeding type ranges from 42.1 ± 2.4 to 62.4 ± 3.0 Bq/kg (wwt). The gross beta activity of species that belong to carnivorous and omnivorous feeding types is insignificantly different. However, the lowest and highest gross beta activities are observed in species that belong to the carnivorous feeding type with 10.6 ± 0.4 Bq/kg (wwt) for bigfin reef squid (*Sepioteuthis lessoniana*) as well as 19.1 ± 0.8 Bq/kg (wwt) for squid (*Teuthida*) and 68.8 ± 2.8 Bq/kg (wwt) for narrow-barred Spanish mackerel (*Scomberomorus commerson*). In general, the highest beta activity was measured in two carnivorous species, namely, 68.8 ± 2.8 Bq/kg (wwt) for narrow-barred Spanish mackerel (*Scomberomorus commerson*) and 65.5 ± 3.4 Bq/kg (wwt) for tuna (*Thunnini*). These species were regarded as large fish. In addition, these fishes are found at the top of the aquatic food chain (Fig. 1), are important sources of food for humans (Aközcan and Uğur 2013), and tend to accumulate a great amount of toxicity and radiation (Milenkovic et al. 2019).

The annual effective dose received by an adult due to consumption of seafood was calculated using the following formula:

3 $$ {\mathrm{DR}}_{\mathrm{r}}={\mathrm{G}}_{\mathrm{r}}\times {\mathrm{CI}}_{\mathrm{r}}\times {\mathrm{DC}}_{\mathrm{r}} $$

where *DR*_*r*_ denotes the annual effective dose (μSv/y), *G*_*r*_ represents the gross α or gross β activity (mBq/l), *CI*_*r*_ stands for the amount of seafood consumed in 1 year (kg), and *DC*_*r*_ is the dose conversion coefficient (Sv/Bq). According to the National Institute of Nutrition in Vietnam, the consumption of seafood in Vietnam is increasing year by year with an average of 18.8 kg/year eaten by adults (Ministry of Health 2010). Following the annual dose conversion factors issued by the World Health Organization (WHO 2017), the annual dose conversion factors used to calculate the annual effective dose of different sources of radionuclides were as follows: ^238^U = 4.5 × 10^−8^ Sv/Bq, ^235^U = 4.7 × 10^−8^ Sv/Bq, ^234^U = 4.9 × 10^−8^ Sv/Bq, ^226^Ra = 2.8 × 10^−7^ Sv/Bq, ^210^Po = 1.2 × 10^−6^ Sv/Bq, ^210^Pb = 6.9 × 10^−7^ Sv/Bq (WHO 2017), and ^40^K = 6.2 × 10^−9^ Sv/Bq (ICRP 2012). Based on research by Carvalho et al. (2011) concerning deep sea fish and other organisms, the authors calculated the percentages of ^238^U, ^235^U, ^234^U, ^40^K, ^210^Pb, ^210^Po, and ^226^Ra in the gross alpha and gross beta activities. The calculated results showed that the percentages of ^238^U, ^235^U, ^234^U, ^226^Ra, and ^210^Po were 1.7, 0.1, 1.7, 17.9, and 78.6% in terms of gross alpha activity, respectively, and for ^210^Pb and ^40^K, 0.1 and 99.9% in terms of gross beta activity, respectively. Some artificial radionuclides, e.g., ^134^Cs and ^137^Cs, only emit gamma radiation; moreover, the activities of ^239^Pu, ^240^Pu, ^241^Pu, ^90^Sr, and ^241^Am are very low when compared with natural alpha and beta radiation. Therefore, when using Formula 3 to calculate the annual effective dose, those isotopes can be neglected (Table 3).

**Table 3:** Annual effective dose (μSv/y) of radiation source in seafood for adults

English name	U-238*	U-235*	U-234*	Ra-226*	Po-210*	Pb-210^#^	Total
Groupers	0.31 ± 0.02	0.01 ± 0.00	0.34 ± 0.03	20.4 ± 1.6	383 ± 30	0.53 ± 0.02	405 ± 32
Tuna	0.87 ± 0.10	0.04 ± 0.00	0.95 ± 0.11	57.6 ± 6.4	1082 ± 121	0.68 ± 0.04	1142 ± 127
Shortfin scad	0.35 ± 0.03	0.01 ± 0.00	0.38 ± 0.03	22.8 ± 1.9	429 ± 35	0.40 ± 0.02	453 ± 37
Squid	0.61 ± 0.07	0.03 ± 0.00	0.67 ± 0.07	40.6 ± 4.5	763 ± 85	0.20 ± 0.01	805 ± 90
Bigfin reef squid	0.15 ± 0.02	0.01 ± 0.00	0.16 ± 0.02	9.6 ± 1.4	181 ± 27	0.11 ± 0.00	191 ± 28
Narrow-barred Spanish mackerel	0.19 ± 0.01	0.01 ± 0.00	0.21 ± 0.01	12.6 ± 0.7	236 ± 12	0.72 ± 0.03	250 ± 13
Silver pomfret	0.33 ± 0.03	0.01 ± 0.00	0.37 ± 0.03	22.1 ± 1.7	415 ± 32	0.41 ± 0.02	438 ± 34
Indian mackerel	0.42 ± 0.03	0.02 ± 0.00	0.46 ± 0.04	28.0 ± 2.3	527 ± 43	0.65 ± 0.03	557 ± 45
Lyrate hard clam	0.84 ± 0.08	0.04 ± 0.00	0.92 ± 0.08	55.2 ± 5.0	1037 ± 94	0.50 ± 0.02	1095 ± 99
Granular ark /blood cockle	1.05 ± 0.12	0.04 ± 0.00	1.15 ± 0.13	69.1 ± 7.6	1298 ± 144	0.44 ± 0.03	1370 ± 152
Giant tiger prawn	0.62 ± 0.08	0.03 ± 0.00	0.68 ± 0.09	41.3 ± 5.2	775 ± 98	0.38 ± 0.02	818 ± 103
Average	0.52	0.02	0.57	34.5	647	0.46	684

*The annual effective dose values and uncertainties in this table are estimations based on gross alpha and gross beta values, not measured individual activity concentrations

**Fig. 2:**
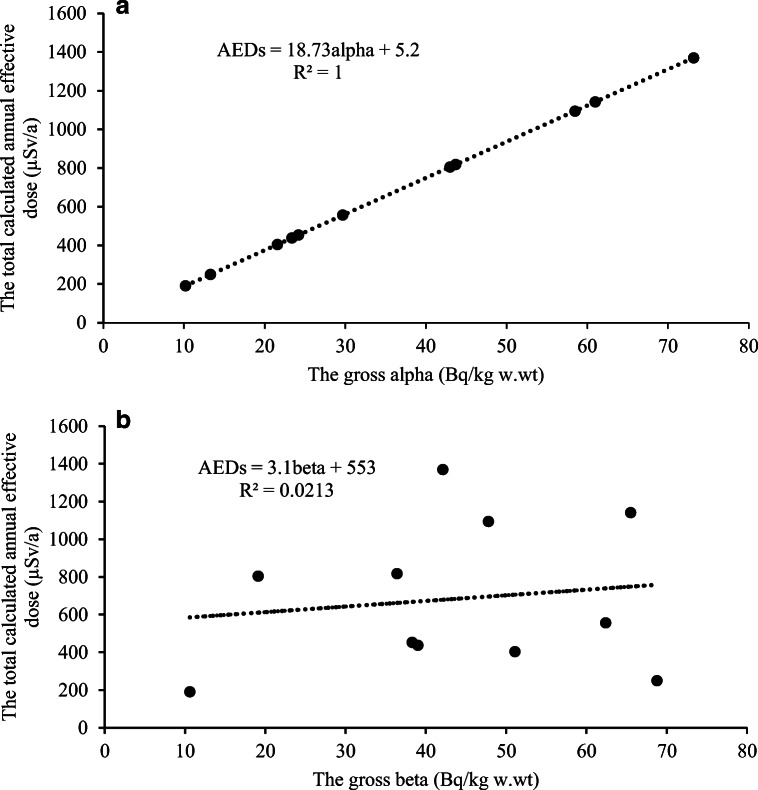
**a**The relationship between the total annual effective dose and gross alpha activity. **b** The relationship between the total annual effective dose and gross beta activity

It must be noted that the presented total annual effective doses are estimates and the isotopes were not measured separately. ^210^Po contributes the largest proportion of activity to the average annual effective dose calculated due to its digestion in seafood by adults, contributing from 181 to 1298 μS/y (647 μS/y on average), while ^235^U contributes the smallest proportion of activity which ranges from 0.01 to 0.04 μS/y (0.02 μS/y on average). The order of contribution to the annual effective dose in descending order is ^210^Po, ^226^Ra, ^40^K, ^234^U, ^238^U, ^210^Pb, and ^235^U. The contribution to the annual effective dose for adults of the β radiation source ^210^Pb is 0.46. The gross alpha activity is significantly greater than the gross beta. There is a linear relationship between the total annual effective dose and gross alpha activity with *R*^2^ = 1 (strong positive correlation with a Pearson correlation coefficient *R*(11) = 1, *p* < 0.05), while for the gross beta activity, *R*^2^ < 1 (weak positive correlation with a Pearson correlation coefficient *R*(11) = 0.14, *p* = 0.681, not significant) (Figs. 2–b). The total annual effective dose varies from 191 to 1370 μS/y. The highest total annual effective dose belongs to an omnivorous species, blood cockle. The lowest total annual effective dose is associated with a carnivorous species, bigfin reef squid. The total average annual effective dose is 684 μS/y which is less than the annual effective dose limit of Vietnam (TCVN 2008).

**Fig. 3:**
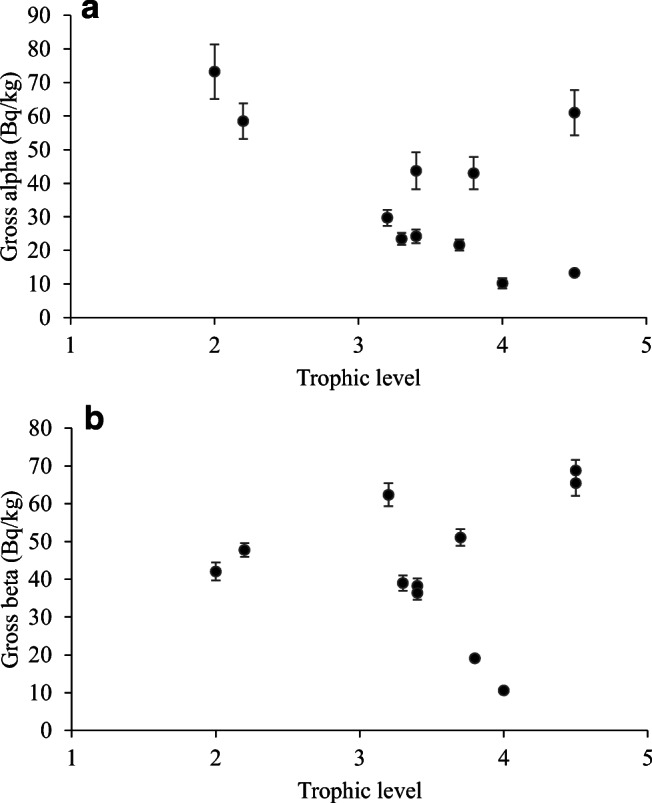
**a** The relationship between trophic level and gross alpha activity. **b** The relationship between trophic level and gross beta activity

Figure 3 and b show the relationship between tropic level and gross alpha and gross beta activity concentration, respectively.

Comparing Fig. 3 and b, lower trophic levels tend to have higher gross alpha activity concentration (moderate negative correlation according to the Pearson correlation coefficient, *R*(11) = − 0.55, *p* = 0.086), while this is not the case for gross beta activity concentration (weak positive correlation, *R*(11) = 0.12, *p* = 0.717); however, neither relationship is considered significant. The relationship might be influenced by the bivalve mollusks accumulating contaminants. It should be noted that gross alpha and beta activity not only depend on feeding habits, the rate of metabolism, and the environment but also on the size of the species, its age, and other parameters, e.g., the depth and temperature of the water.

## Conclusion

A study of gross alpha and gross beta activities in Vietnamese seafood is presented for the first time. The results particularly showed that the total annual effective dose is principally contributed to by gross alpha activity. Among these, the activity concentration of ^210^Po contributed over 70% in total while that of ^235^U was the smallest. The increase in annual effective dose is most strongly associated with ^210^Po, followed by ^226^Ra among other radionuclides. The smallest annual effective dose belongs to the uranium isotopes and ^210^Pb. It must be noted that this is based on the ratio of radionuclides in fish from Carvalho et al. (2011) and gross alpha and beta measurements, not individual radionuclide concentrations.

Of the species studied, the carnivorous bigfin reef squid (*Sepioteuthis lessoniana*) exhibited the lowest gross alpha activity, while the omnivorous bottom feeder, blood cockle (*Anadara granosa*), presented the highest gross alpha activity. With regard to gross beta activity, both the lowest and highest activities were exhibited by carnivorous species, namely, bigfin reef squid (*Sepioteuthis lessoniana*), squid (*Teuthida*), and narrow-barred Spanish mackerel (*Scomberomorus commerson*), respectively.
